# Plasma TNFSF13B and TNFSF14 Function as Inflammatory Indicators of Severe Adenovirus Pneumonia in Pediatric Patients

**DOI:** 10.3389/fimmu.2020.614781

**Published:** 2021-01-19

**Authors:** Huifeng Fan, Bingtai Lu, Can Cao, Hui Li, Diyuan Yang, Li Huang, Tao Ding, Minhao Wu, Gen Lu

**Affiliations:** ^1^ Department of Respiration, Guangzhou Women and Children’s Medical Centre, Guangzhou Medical University, Guangzhou, China; ^2^ Guangzhou Institute of Pediatrics, Guangzhou Women and Children’s Medical Centre, Guangzhou Medical University, Guangzhou, China; ^3^ School of Life Sciences, Sun Yat-sen University, Guangzhou, China; ^4^ Department of Immunology, Zhongshan School of Medicine, Sun Yat-sen University, Guangzhou, China; ^5^ Pediatric Intensive Care Unit, Guangzhou Women and Children’s Medical Center, Guangzhou Medical University, Guangzhou, China

**Keywords:** tumor necrosis factor receptor superfamily (TNFRSF), adenovirus, pediatric, pneumonia, inflammation

## Abstract

**Background:**

Human adenoviruses (HAdV) infection caused pneumonia remains a major threat to global children health. Currently, diagnosis of severe HAdV pneumonia in children is hampered by the lack of specific biomarkers. Also, the severity of adenovirus pneumonia in pediatric patients is generally based on clinical features and existing biomarkers do not reliably correlate to clinical severity. Here, we asked whether local and systemic inflammatory mediators could act as biomarkers predicting severe HAdV pneumonia in children.

**Methods:**

Totally 37 common inflammatory protein levels were determined by Luminex assay in plasma and bronchoalveolar lavage (BAL) from pediatric patients who were diagnosed with HAdV pneumonia, and their correlation with the disease severity and lung lesion were assessed using statistical and bioinformatic analysis.

**Results:**

Among 37 inflammatory cytokines, the protein levels of 4 TNF superfamily (TNFSF) members and their receptors (TNF receptor superfamily, TNFRSF) [TNFSF13B, TNFSF14, sTNF-R1 and sTNF-R2] in the plasma and 7 TNFSF/TNFRSF members [TNFSF12, TNFSF13, TNFSF13B, TNFSF14, TNFRSF8, sTNF-R1, and sTNF-R2] in the BAL were enhanced in patients with HAdV pneumonia compared with control subjects with airway foreign body. Moreover, the protein levels of all the tested TNFSF/TNFRSF members (except TNFSF12) were elevated in the BAL of severe group compared with non-severe HAdV pneumonia patients, while only TNFSF13B and TNFSF14 were dramatically increased in the plasma of severe cases, and positively related to the plasma CRP levels. In addition, ROC analysis indicated that TNFSF13B and TNFSF14 displayed a great potential to predict severe HAdV pneumonia.

**Conclusion:**

In pediatric HAdV pneumonia, TNFSF/TNFRSF members function as key molecules in local and systemic inflammatory network, and the plasma TNFSF13B and TNFSF14 may be the potential local and systemic inflammatory indicators of severe HAdV pneumonia in pediatric patients.

## Introduction

As an important family of DNA viruses, human adenoviruses (HAdV) cause approximately 2%–5% of the respiratory tract infections and 4%–10% of all types of pneumonia (1, 2). Outbreaks of HAdV do not occur at specific times of the year, but can lead to widespread transmission and serious adverse outcomes in neonatal intensive care units (NICUs) (3). About 14%–60% of HAdV infected patients have a risk to develop long-term respiratory complications, such as post-infectious bronchiolitis obliterans (PIBO) and bronchiectasis (4).

Although World Health Organization (WHO) has raised general danger signs for Integrated Management of Neonatal and Childhood Illnesses (IMCI), which provides a rough guide for hospitalization (5), there is no validated clinical scoring system to predict the need of hospitalization for pediatric patients (6). However, clinically, early evaluation and prediction of HAdV pneumonia remains a big challenge, due to the lack of sensitive and specific indicators. Therefore, early recognition of severe cases is important for decreasing the morbidity and improving the prognosis.

Previous study has confirmed that HAdVs can cause lytic infection in epithelial cells and result in acute respiratory diseases. More importantly, virions can be recognized by various pattern recognition receptors on host cells, and therefore induce host inflammation, or even immune pathological damage, if the inflammatory response is out of control (7). Intensive efforts have been motivated to explore the underlying mechanisms of adenovirus-induced innate immunity, and how to balance the inflammatory response for viral elimination and tissue injury. Nonetheless, whether those inflammatory markers identified in the serum can reflect the local lung inflammation remains unclear.

The tumor necrosis factor (TNF) superfamily (TNFSF) is a protein superfamily of Type II transmembrane proteins commonly containing the TNF homology domain. So far, more than 20 TNFSF members have been identified, each can bind to their corresponding receptors that belong to TNF receptor superfamily (TNFRSF), and play critical roles in mediating cell apoptosis, proliferation, activation and differentiation (8). Moreover, several TNFSF/TNFRSF members also have their soluble forms that function like cytokines to modulate diverse biological processes, especially the anti-infection immunity and inflammatory response (9).

Studies have demonstrated that several soluble TNFSF/TNFRSF members are involved in respiratory diseases or disorders. For example, TNF functions to activate macrophages and to induce Th1 response, and therefore is important in host anti-mycobacterial defense (9). Furthermore, increased TNFSF8 and TNFRSF8 levels are detected in the serum of patients with asthma (10) and chronic inflammatory diseases (11). It is also reported that TNFSF14 (also called LIGHT) is involved in dysregulated mucosal function or pulmonary diseases, such as pneumonia, non-small cell lung cancer (12), asthma and lung fibrosis (13). TNFSF14 can bind to its receptors, lymphotoxin receptor (LTR) or herpesvirus entry mediator (HVEM, also called TNFRSF14), and then stimulate T cells and innate immune response (14). During acute respiratory virus infection, TNFSF14 promotes the generation of circulating and lung-resident memory CD8^+^ T cells (15) and its expression is enhanced in neutrophils and macrophages of HAdV55 infection patients vs controls (16). A recent study has reported that TNFSF14 is upregulated in serum of hospitalized COVID-19 patients with cytokine release syndrome and acute respiratory distress syndrome (ARDS) (17). In addition, TNFSF13B (also called BAFF), which can influence B cell maturation, proliferation and antibody class switch (18), is also implicated in the pathophysiology of pulmonary diseases. High TNFSF13B level is detected in the bronchoalveolar lavage fluid of patients with idiopathic pulmonary fibrosis and increased in the airway during respiratory syncytial virus (RSV) infections (19). Excessive expression of TNFSF13B in chronic obstructive pulmonary disease (COPD) leads to aggravated lung inflammation and alveolar wall destruction (20).

At present, the differences between host systemic and local inflammation, and which inflammatory indicators is closely related to the severity of HAdV pneumonia still need further investigation. In this regard, the present study is designed to explore the systemic and local inflammatory response during HAdV pneumonia, which may provide certain clues of inflammatory biomarkers to predict the progression of severe HAdV pneumonia.

## Materials and Methods

### Design and Study Participants

We evaluated 566 pediatric patients who were diagnosed with HAdV pneumonia in Guangzhou Women and Children’s Medical Center from December 2017 to December 2018, according to the evidence-based guidelines regarding the diagnosis of pneumonia in children published by WHO (21). Briefly, the evidence of HAdV infection was confirmed by positive multiplex polymerase chain reaction (PCR) for HAdV from lower respiratory tract samples, including valid sputum and BAL fluid. Viral DNA was extracted and the seven hypervariable regions (HVRs) of HAdV hexon gene were amplified by PCR. A total of 13 HVR sequences from prototype and circulating strains of HAdV-B in China were used for genotyping analysis, and five HAdV strains were tested in the present study, including (for reference, the names include the corresponding GenBank accession number, type, and strain name): AY599834_HAdV-3_GB, DQ099432_HAdV-3_Guangzhou01, Y594255_HAdV-7_Gomen, KC440171_HAdV-7_DG01, AY601633_HAdV-21_AV-1645.

**Figure 1:**
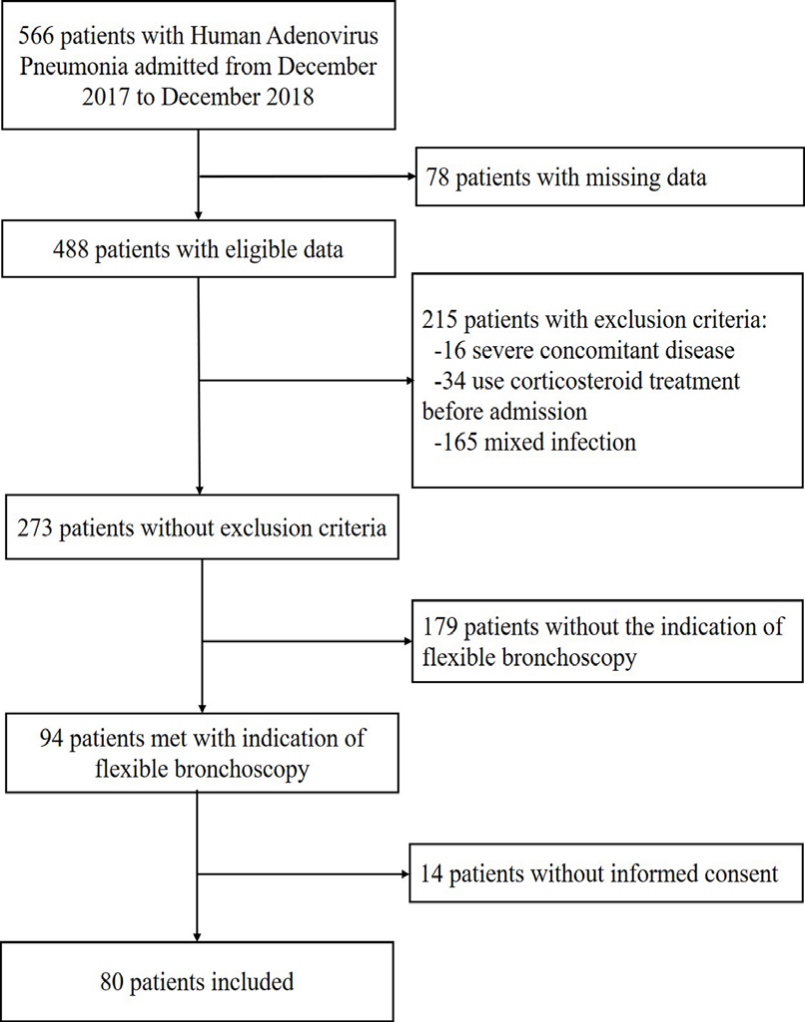
Inclusion criteria of the study cohort of pediatric human adenovirus (HAdV) pneumonia.

A total of 80 patients meeting the inclusion criteria were enrolled in the present study (as shown in **Figure 1**). In addition, 20 pediatric patients with foreign body in trachea were selected as control subjects, who were verified without recent respiratory infections by clinical characteristics and image manifestations. Written informed consent was obtained from all the participants’ guardians, and then the participants’ BAL fluid and peripheral blood samples as well as their clinical data, including the laboratory and radiology findings, were collected.

Exclusion criteria were mixed infections, known or suspected active tuberculosis, severe concomitant disease (chronic pulmonary disease except asthma, severe cardiovascular disease, neoplasia, and kidney or liver disease), primary immunodeficiency, acquired immunodeficiency syndrome and immunosuppressive medications before admission. All the plasma and BAL specimens as well as clinical examination were collected or performed at the acute stage of infection, before corticosteroid treatment.

Severe or non-severe HAdV pneumonia was classified on the basis of clinical features. The diagnosis of severe cases was obtained when the following criteria were fulfilled (22): 1) major criteria: invasive mechanical ventilation; fluid refractory shock; acute need for noninvasive positive pressure ventilation; and hypoxemia requiring fraction of inspired oxygen(FiO_2_) greater than the inspired concentration or flow feasible in the general care area; 2) minor criteria: respiratory rate greater than the WHO classification for age; apnea; increased work of breathing (e.g. retractions, dyspnea, nasal flaring, and grunting); PaO_2_/FiO_2_ ratio <250; multilobar infiltrates; Pediatric Early Warning Score >6; altered mental status; hypotension; presence of effusion; comorbid conditions (e.g., hemoglobin SS disease, immunosuppression, and immunodeficiency); and unexplained metabolic acidosis.

This study was conducted in accordance with the declaration of Helsinki and approved from the Ethics Committee of Guangzhou Women and Children’s Medical Center, Guangzhou Medical University. The study was conducted in accordance with the Strengthening the Reporting of Observational Studies in Epidemiology (STROBE) guidelines.

### Flexible Bronchoscopy

Flexible bronchoscopy was performed for children with HAdV pneumonia who met with the following criteria: extensive pulmonary infiltrates or consolidation with high-resolution CT (HRCT) findings. All bronchoscopies were clinically indicated and were performed under general anesthesia with a flexible bronchoscope. Usually bronchoalveolar lavage (BAL) was carried out in the most-affected area (identified radiologically and/or endoscopically) and using normal sterile saline previously warmed to body temperature (37°C). The protocols for BAL were performed by instilling three or five fractions of the same volume (5–10 ml) each lobe according to the weight and age of child (BAL volume to body weight about 3 ml/kg). The recovery volume of BAL is more than 40% was acceptable.

### Multiplex Immunoassay

Totally 37 inflammatory proteins in the plasma and BAL supernatant was measured using “Bio-Plex Pro™ Human Cytokine Standard 37-Plex, Group I-kit” from BioRad with magnetic bead-based multiplex immunoassay for Luminex (LX1000), according to the instructions of the manufactures. For results under the limits of detection (LOD), the lowest value of the standard for each of the cytokines was used for statistics analysis. The 37 inflammatory proteins consist of tumor necrosis factor (TNF) superfamily [TNFRSF8, TNFSF12, TNFSF13, TNFSF13B, TNFSF14], interleukin (IL) family [IL-2, IL-6, IL-8, IL-10, and IL-11, IL-12 p40, IL-12 p70, IL-19, IL-20, IL-22 and IL-26, IL-27 p28, IL-32, IL-34, IL-35], interferon family (IFN) [IFN-α2, IFN-β, IFN-γ, IFN-λ2, and IFN-λ1], matrix metalloproteinases family (MMP)[MMP-1, MMP-2, and MMP-3], and soluble receptors [sTNF-R1, sTNF-R2, sIL-6Rα and sIL-6Rβ, sCD163], as well as other cytokines [Chitinase 3-like 1, Pentraxin-3, thymic stromal lymphopoietin (TSLP), Osteocalcin (OCN), Osteopontin (OPN)].

### Statistical and Bioinformatics Analysis

Due to the skewed distribution of inflammatory protein levels in plasma and BAL, medians with ranges were used to express summarized data. The nonparametric Mann-Whitney rank sum test was used for two-group analysis of continuous variables, and Kruskal-Walls test was used for three-group analysis of continuous variables. Categorical variables were assessed using the χ^2^ test. Receiver operating characteristic (ROC) analysis was performed to assess the sensitivity and specificity of TNFSF13B and TNFSF14 as biomarker and was expressed as Area Under the Curve (AUC). A *P* value of <0.05 was considered to be statistically significant. Data were considered significant at ^∗^ *P*<0 05; ^∗∗^ *P*<0.01; ^∗∗∗^ *P*<0.001, and ^∗∗∗∗^ *P*<0.0001.

Data were analyzed by GraphPad software (Prism 8.0) and visualized by ggplot2 package of R software (version 3.6.1). The differentially expressed inflammatory proteins were analyzed by Cytoscape (http://www.cytoscape.org), which is an open source software platform for visualizing molecular interaction networks and biological pathways, and integrating these networks with annotations.

### Description of the Cohort

In the present study, 80 pediatric patients with newly diagnosed HAdV pneumonia and 20 control subjects with foreign body in trachea were included following the admission criteria as shown in **Figure 1**. The clinical features, laboratory examination, as well as treatment and outcome characteristics of each patients were displayed in **Table 1**.

**Table 1:** Characteristics of pediatric patients with human adenovirus (HAdV) pneumonia enrolled in the study.

Variable	Control cases	Non-severe cases	Severe cases	*P* value
	(n = 20)	(n = 41)	(n = 39)	
**Demographic**				
Age (months), median(range)	35.50 (6–108)	40.50(7–120)	24(6–120)	0.3754*^a^*
Gender, male (%)	9((45.00)	25(60.98)	18(46.15)	0.3247*^a^*
**Signs and Symptoms**				
Fever, No. (%)		41(100.00)	39(100.00)	1.0000
The duration of fever(days), median(range)		8(0–17)	14(5–24)	**<0.0001**
Wheezing, No. (%)		6(14.63)	12(30.77)	0.1104
Dyspnea, No. (%)		0(0.00)	39(100.00)	**<0.0001**
Change in level of consciousness, No. (%)		0(0.00)	19(48.72)	**<0.0001**
Digestive symptoms, No. (%)		4(9.76)	20(51.28)	**<0.0001**
**Laboratory findings** *^b^*				
White blood counts (×10^9/L)(5–12), median(range)		8.35(3–35.8)	9.2(2.1–28.7)	0.7237
Neutrophil(×10^9/L)(5–12), median(range)		3.195(0.32–25.51)	4.38(0.98–16.71)	0.6549
Hemoglobin (g/L)(105–145),median(range)		113.5(79–138)	104.5(72–145)	**0.0005**
Platelet (×10^12/L)(140–440), median(range)		325(89–914)	337(70–1122)	0.8047
C-reactive protein (mg/L)(<5), median(range)		0.78(0.2–98.4)	28.10(0.26–282.65)	**<0.0001**
Lactate dehydrogenase (U/L) (159–322), median(range)		264(167–327)	402(172–1444)	**0.0002**
Albumin (g/L)(40–50), median(range)		38.4(28.8–47.2)	34.35(23.6–46.2)	**0.0002**
PaO_2_(kPa)(≥10.9), median (range)		9.79(8.97–11.96)	8.1(5.2–12.36)	**0.0081**
PaCO_2_(kPa)(4.66–5.99), median (range)		4.65(1.9–6.01)	4.9(3.61–7.58)	0.3584
HAdV-3, No. (%)		2(4.88)	5(7.70)	0.2578
HAdV-7, No. (%)		0(0.00)	30(76.92)	**<0.0001**
**Radiology** *^c^*				
Consolidation(>1 lobe), No. (%)		8(19.51)	37(94.87)	**<0.0001**
Hydrothorax, No. (%)		0(0.00)	9(23.08)	**0.0009**
Mosaic sign, No. (%)		0(0.00)	10(25.64)	**0.0004**
**Treatment**				
Antibotics, No. (%)		5(12.20)	18(46.15)	**0.0011**
Immunoglobulin, No. (%)		4(9.75)	38(97.44)	**<0.0001**
Systemic corticosteroid, No. (%)		9(21.95)	25(64.10)	**0.0002**
Mechanical ventilation, No. (%)		0(0.00)	8(20.51)	**0.0021**
**Outcomes**				
Length of stay(days), median(range)		7(4–17)	15.5(7–28)	**<0.0001**
Mortality, No(%)		0(0.00)	1(2.56)	0.9999

a p values are two-sided and were adjusted by Bonferroni method for multiple testing.

b The data of Laboratory findings were collected from patients with acute exacerbation.

c Judged by chest radiograph or CT scan in whole course of the patients. The values in bold represent that the difference between the two was statistically significant (P < 0.05).

Age and gender between the HAdV pneumonia group and the control group displayed no statistically significant difference(*P*>0.05). In patients underwent BAL, 39 were confirmed severe cases (39/80, 48.75%). All the patients had fever in different degree, but the fever duration was significantly longer in severe group (*P*<0.05). Furthermore, C-reaction protein (CRP) and lactate dehydrogenase (LDH) levels were significantly elevated, while albumin (ALB) and PaO_2_ were reduced, in severe vs non-severe patients (*P*<0.05). Thirty children were confirmed with HAdV-7 in severe group, which had a higher proportion compared to non-severe group(*P*<0.05). Consolidation was the common imaging finding in all enrolled patients (80/80, 100%) on HRCT. In 56.25% (45/80) of patients, we observed more than one lobe of lung consolidation. Thirty-eight children were given gamma globulin in severe group (97.44%), and systemic corticosteroids were used for 25 severe patients (64.10%). Moreover, 8 severe cases were treated by mechanical ventilation (20.51%). Only one child died from this disease.

## Results

### The Expression of Inflammation-Related Proteins in Pediatric Patients With Human Adenovirus Pneumonia

To investigate the inflammatory protein profile of children HAdV pneumonia, we examined the protein levels of 37 common inflammatory molecules in plasma and BAL of children with recently diagnosed HAdV pneumonia (**Table 2**). In the plasma, the protein levels of 24 inflammatory molecules were upregulated in the HAdV pneumonia patients compared with the control group, including TNFSF13B, TNFSF14, sTNF-R1, sTNF-R2, IFN-α2, IFN-γ, sIL-6Rα, sIL-6Rβ, IL-10, IL-12 p70, IL-20, IL-22, IL-26, IL-32, IL-34, IL-35, MMP-1, MMP-2, MMP-3, OPN, Pentraxin-3, TSLP, sCD130, and Chitinase 3-like 1 (*P*<0.05). The plasma levels of IFN-λ2 and IL-19 were downregulated in HAdV pneumonia. In addition, the protein levels of seven inflammatory proteins [IFN-λ2, IL-2, IL-20, IL-22, IL-32, IL-34, and IL-35] were comparable in the BAL of HAdV pneumonia patients (*P*>0.05), and other proteins were significantly increased in HAdV pneumonia patients when compared to that in control group (*P*<0.05).

**Table 2:** Plasma and BALF cytokines profiles in human adenoviruses (HAdV) pneumonia patients and control subjects.

Cytokines^a^	Plasma			BAL fluid		
	Control^b^	HAdV pneumonia^b^	*P* value	Control^b^	HAdV pneumonia^b^	*P* value
TNFRSF8	1,098.86(608.32–2,241.03)	1,137.14(35.29–2,431.02)	0.9932	2.02(2.02–18.47)	43.31(2.02–872.13)	**<0.0001**
TNFSF12	299.59(211.12–413.26)	250.5(23.77–802.63)	0.2331	5.20(0.04–97.90)	72.92(0.81–531.56)	**0.0037**
TNFSF13	33,715.22(20,145.35–47,211.27)	32,409.875(815.23–343,035.74)	0.4550	295.91(295.91–1,684.39)	15,199.40(232.47–343,035.74)	**<0.0001**
TNFSF13B	4,555.35(2,850.35–9,719.58)	10,993.02(4,577.61–113,929.27)	**<0.0001**	276.84(106.12–792.16)	9,013.66(106.12–171,571.07)	**<0.0001**
TNFSF14	0.52(0.52–0.68)	8.44(0.71–38.35)	**<0.0001**	0.71(0.71–6.76)	7.11(0.71–53.47)	**<0.0001**
sTNF-R1	776.55(182.66–1,626.93)	1,440.62(285.58–3,621.77)	**<0.0001**	88.04(0.09–225.06)	2,306.53(0.09–18,022.77)	**<0.0001**
sTNF-R2	392.00(109.66–984.74)	527.865(94.38–4,622.34)	**0.0144**	2.42(2.42–23.04)	603.43(2.42–5,033.70)	**<0.0001**
IFN-α2	1.48(1.48–72.67)	2.39(1.48–97.83)	**0.0272**	1.48(1.48–1.98)	2.39(1.48–4,310.32)	**0.0006**
IFN-β	35.51(18.11–65.60)	48.07(0.43–165.43)	0.3210	0.34(0.34–13.85)	13.09(0.34–90.58)	**0.0002**
IFN-γ	91.185(13.03–176.43)	10,421.92(1.93–113,929.27)	**<0.0001**	1.93(1.93–106.12)	16.63(1.93–48,838.57)	**0.0079**
IFNλ2	14.33(6.29–123.58)	7.28(6.29–56.19)	**0.0008**	7.28(7.28–7.30)	7.28(6.29–615.64)	0.7497
IFNλ1	94.365(4.24–214.34)	139.325(4.24–567.31)	0.2443	4.24(4.10–4.24)	14.47(4.24–161.3)	**0.0002**
IL-2	0.20(0.20–0.21)	0.21(0.20–0.21)	0.1691	0.20(0.20–0.21)	0.21(0.20–0.21)	0.4337
sIL-6Ra	630.16(240.97–1,736.17)	1,533.09(108.46–3,156.89)	**<0.0001**	9.74(6.36–194.29)	239.92(6.36–2,239.35)	**<0.0001**
sIL-6Rb	16,397.71(2,954.46–32,444.81)	28,862.72(3,381.98–57,118.16)	**0.0002**	29.31(2.42–1286.75)	4,570.37(12.13–11,733.08)	**<0.0001**
IL-8	8.22(5.12–103.59)	24.785(5.12–860.20)	0.3097	5.12(5.12–12.01)	1,561.65(5.12–716,370.00)	**<0.0001**
IL-10	0.31(0.31–4.1)	1.80(0.31–24.99)	**<0.0001**	0.31(0.31–5.06)	2.62(0.31–166.69)	**0.0016**
IL-11	1.405(0.62–3.22)	1.17(0.09–8.07)	0.6753	0.09(0.09–0.13)	0.23(0.09–6.54)	**<0.0001**
IL-12 p40	23.295(0.18–57.19)	21.14(0.18–160.55)	0.9019	0.18(0.18–9.62)	1.93(0.18–36.01)	**0.0036**
IL-12 p70	0.04(0.00–0.04)	0.04(0.04–0.91)	**<0.0001**	0.35(0.35–0.93)	0.35(0.04–1.81)	**0.0034**
IL-19	51.64(23.42–86.07)	6.30(0.85–84.66)	**<0.0001**	0.85(0.85–19.68)	23.45(0.85–305.14)	**0.0002**
IL-20	0.07(0.06–0.07)	0.07(0.07–1.28)	**0.0048**	0.07(0.07–0.10)	0.07(0.07–0.09)	0.4337
IL-22	0.07(0.07–0.10)	2.63(2.42–54.65)	**<0.0001**	2.63(2.63–12.56)	15.92(2.42–68.28)	0.0896
IL-26	0.18(0.18–5.40)	0.71(0.18–33.72)	**<0.0001**	0.18(0.18–8.76)	0.93(0.18–27.09)	**<0.0001**
IL-27 p28	10.81(4.00–39.08)	1.06(0.81–82.13)	0.1707	0.81(0.81–9.43)	3.46(0.81–25.70)	**0.0062**
IL-32	0.30(0.20–0.30)	24.25(0.30–400.76)	**<0.0001**	54.37(0.3–236.73)	105.25(0.30–622.39)	0.1226
IL-34	7.23(7.23–7.35)	26.81(7.23–26.81)	**<0.0001**	7.23(7.23–196.74)	26.81(7.23–206.16)	0.2451
IL-35	40.33(11.52–242.16)	104.20(1.22–358.17)	**0.0176**	5.36(5.36–97.71)	16.98(1.22–128.25)	0.0666
MMP-1	1,344.02(681.28–2,107.99)	2,232.475(66.94–4,754.74)	**0.0218**	66.94(66.94–582.60)	329.14(66.94–2,302.43)	**0.0002**
MMP-2	81,966.14(36,760.82–151,785.20)	127,257.86(5,168.95–395,274.55)	**0.0002**	290.16(46.52–1,497.66)	3,267.16(46.52–30,427.41)	**<0.0001**
MMP-3	1,964.71(1,072.78–3,433.51)	3,803.15(419.14–11,349.84)	**<0.0001**	117.39(68.27–907.57)	476.40(68.27–3,349.45)	**<0.0001**
OCN	8,640.87(2,164.22–12,265.02)	8,763.41(9.53–29,622.58)	0.8314	82.88(81.09–82.88)	51.64(7.05–274.47)	**0.0059**
OPN	23,263.95(14,154.72–38,531.64)	56,586.63(1,121.27–150,998.40)	**<0.0001**	224.72(147.71–513.41)	2,551.08(186.36–58,975.46)	**<0.0001**
Pentraxin-3	1,101.89(483.88–3,642.62)	2,187.57(0.23–8,117.78)	**<0.0001**	0.85(0.85–12.94)	128.47(0.23–2,449.29)	**0.0012**
TSLP	48.24(27.47–79.98)	72.425(0.19–212.72)	**0.0175**	0.19(0.19–12.43)	9.52(0.19–51.05)	**<0.0001**
sCD163	36,976.80(24,217.71–83,977.63)	77,228.775(16,913.64–1,743,400.00)	**<0.0001**	6,466.72(1,009.35–23,884.29)	92,593.82(1,009.35–4,019,200.00)	**<0.0001**
Chitinase 3like 1	2,424.37(1,486.18–4,289.79)	3,676.99(1,036.60–7,747.00)	**<0.0001**	30.03(30.03–581.24)	3,395.01(30.03–6,816.59)	**<0.0001**

a For all the cytokines tested, the unit is pg/ml and data were shown as median(range).

b Totally 80 patients with HAdV pneumonia and 20 control subjects were enrolled. The values in bold represent that the difference between the two was statistically significant (P < 0.05).

**Figure 2:**
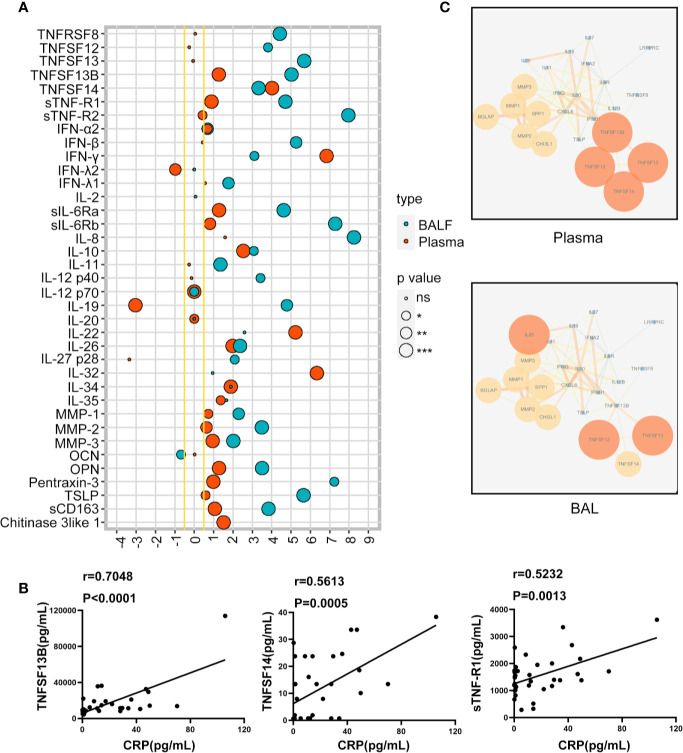
The expression of inflammation related proteins in pediatric patients with human adenovirus (HAdV) pneumonia. The lollipop graphs depict the log2(Fold Change) of different expressions of cytokines in children plasma and bronchoalveolar lavage (BAL) with HAdV pneumonia compared to controls. **(A)**. The correlation between TNFSF13B, TNFSF14, sTNF-R1, and C-reaction protein (CRP) in the plasma of children with HAdV pneumonia (n = 35) **(B)**. Cytokine-cytokine Interaction Networks in children with HAdV pneumonia compared to control group **(C)**. ns, no significance; *P < 0.05; **P < 0.01; ***P < 0.001; ****P < 0.0001.

To explore the key inflammatory markers of children HAdV pneumonia, we analyzed the inflammation related proteins based on the fold change and statistical significance. Among the inflammatory molecules with the most significant statistical differences (*P*<0.0001), most of TNFSF/TNFRSF members (TNFSF12, TNFSF13, TNFSF13B, TNFSF14, TNFRSF8, sTNF-R1, sTNF-R2) were greatly enhanced in the BAL of HAdV pneumonia children (*P*<0.0001) (**Figure 2A**). While only TNFSF13B, TNFSF14 and sTNF-R1 were dramatically enriched in the plasma of HAdV pneumonia patients (*P*<0.0001) (**Figure 2A**). Furthermore, we investigated the relationship between those TNFSF/TNFRSF members and CRP in the plasma, and found a significantly positive correlation between TNFSF13B/TNFSF14/sTNF-R1 and CRP in the plasma (**Figure 2B**).

We also analyzed the inflammatory interaction network in the plasma and BAL of HAdV pneumonia patients using Cytoscape, and the results showed that the most featured inflammatory molecules in the plasma were TNFSF12/13 and IL26, while TNFSF12/13/14 play a key role in the network in BAL of HAdV pneumonia children (**Figure 2C**). Together, these data suggest that TNFSF/TNFRSF members, especially TNFSF13/14, are critical in both local and systemic inflammatory response during HAdV pneumonia.

### The Expression of Tumor Necrosis Factor Superfamily/Tumor Necrosis Factor Receptor Superfamily Members Were Associated With the Disease Severity of Pediatric Human Adenovirus Pneumonia

**Figure 3:**
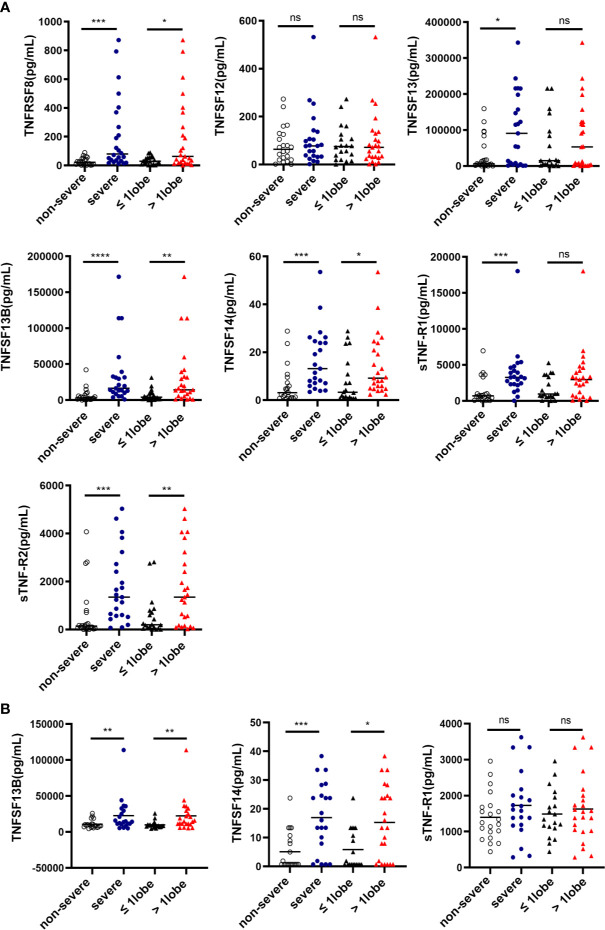
The expression of Tumor Necrosis Factor Superfamily (TNFSF)/TNF receptor superfamily (TNFRSF) members were associated with the disease severity of pediatric human adenovirus (HAdV) pneumonia. Comparisons of the cytokine concentrations of bronchoalveolar lavage (BAL) from children with HAdV pneumonia according to the severity and the consolidation scope **(A)**. Comparisons of the cytokine concentrations of plasma from children with HAdV pneumonia according to the severity and the consolidation scope **(B)**. Data was shown as mean ± SD. ns, no significance; *P < 0.05; **P < 0.01; ***P < 0.001; ****P < 0.0001.

Since both local and systemic inflammation play an important role in the disease severity of HAdV pneumonia, next, we compared the protein levels of TNFSF/TNFRSF members in the plasma and BAL in the non-severe vs severe group of HAdV pneumonia children. In BAL fluid, 6 TNFSF/TNFRSF members were significantly elevated in severe patients compared to non-severe group [TNFSF13, TNFSF13B, TNFSF14, TNFRSF8, sTNF-R1, sTNF-R2] (*P*<0.05) (**Figure 3A**). While in the plasma, only TNFSF13B and TNFSF14 levels were increased in the severe group (*P*<0.05) (**Figure 3B**).

Clinically, the range of consolidated areas in the lungs is an important imaging indicator of the disease severity of HAdV pneumonia, therefore, we classified HAdV pneumonia children to two groups, based on whether the consolidated areas in the lungs are limited to one lobe, or involved more than one lobe, and then analyzed the levels of TNFSF/TNFRSF members in plasma and BAL. Consistently, four TNFSF/TNFRSF members in the BAL were increased in patients with lung lesion over one lobe [TNFSF13B, TNFSF14, TNFRSF8, and sTNF-R2](*P*<0.05) (**Figure 3A**), whereas in the plasma, only TNFSF13B and TNFSF14 levels were greatly elevated in patients with lung lesion over one lobe (*P*<0.05) (**Figure 3B**). Thus, we found that TNFSF13B and TNFSF14 levels from plasma and BAL were increased in severe group of HAdV pneumonia children.

### Plasma TNFSF13B and TNFSF14 May Be Potential Indicator of Severe Cases of Pediatric Human Adenoviruses Pneumonia

**Figure 4:**
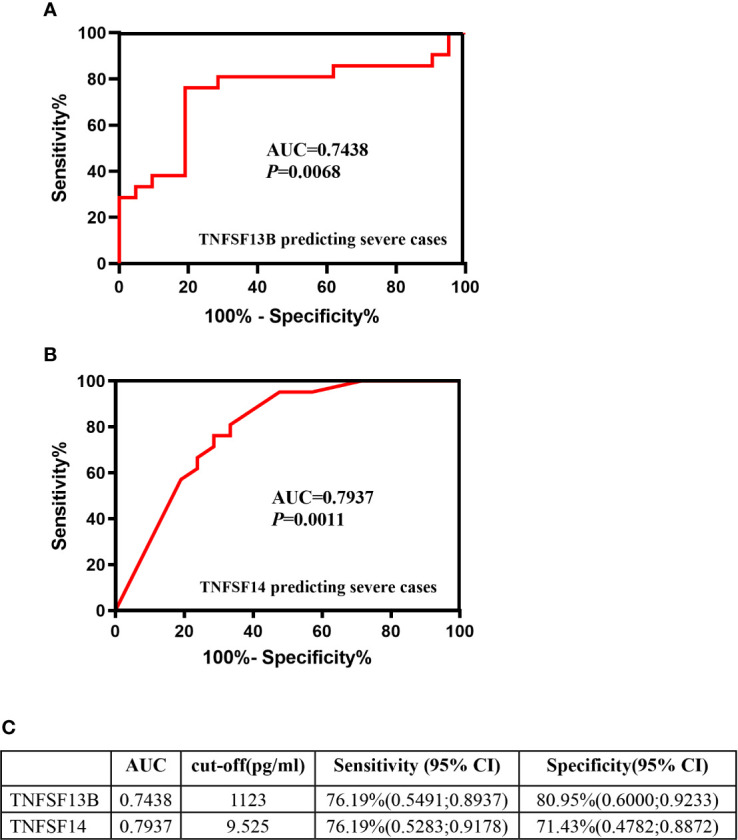
Plasma TNFSF13B and TNFSF14 may be potential indicator of severe cases of pediatric human adenovirus (HAdV) pneumonia. Receiver operating characteristic (ROC) analysis of the ability of TNFSF13B **(A)** and TNFSF14 **(B)** to predict severe HAdV pneumonia. The cut-off value of plasma TNFSF13B and TNFSF14 as well as their sensitivity and specificity in prediction severe cases **(C)**.

The increased protein levels of plasma TNFSF13B and TNFSF14 in severe compared with non-severe cases indicated their potential as systemic inflammatory indicators of severe pediatric HAdV pneumonia. Our data showed that the diagnostic accuracy of plasma TNFSF13B or TNFSF14 in predicting severe cases was 74.38% and 79.37%, respectively (**Figures 4A, B**). Based on the ROC Analysis, when the cut-off value of TNFSF13B was set at 11324 pg/ml, the sensitivity and specificity of severe case prediction was around 76.19% and 80.95%, respectively. While TNFSF14 presented higher accuracy to predict severe cases, yielding a sensitivity of 76.19% and a specificity of 71.43% at the cut-off value of 9.525 pg/ml (**Figure 4C**). The above data indicated that plasma TNFSF13B and TNFSF14 may be potential inflammatory markers in predicting the severe cases of pediatric HAdV pneumonia.

## Discussion

Human adenoviruses (HAdVs) are common causative pathogens of acute respiratory infections in children under 5 years old (23, 24). Most HAdV infections induce self-limiting illness, but approximately 20%–30% of hospitalized children display severe clinical symptoms. So far, there is no specific treatment for HAdV infection (25), and supportive care is mainly used clinically, including hospitalization, intravenous infusion, respiratory support and prevention of secondary infections. If not treated in time, HAdV infections may cause serious sequelae or even death. Therefore, early diagnosis and intervention of severe HAdV pneumonia is important.

It is reported that the severity of lower respiratory tract HAdV infection is closely related to the subtype of HAdV, age of onset, and immune status. However, current diagnosis of severe HAdV pneumonia mainly depends on the clinical symptoms appearing in the late stage of the disease (22, 25), while the specific indicators for early recognition of severe HAdV patients are still lacking. Inflammatory response is a double-edged sword. A moderate inflammatory response is conducive to the removal of the virus, but sustained excessive inflammatory response often cause immune pathological tissue damage, such as ARDS (24). In this regard, the present study was designed to explore the local and systemic inflammatory response in HAdV pneumonia pediatric patients, and identify potential inflammatory indicators as early warning signs of severe cases.

We assessed the protein levels of 37 common inflammatory molecules in the plasma and BAL from children with HAdV pneumonia and control group with airway foreign body using Luminex immunoassay. Among all the tested inflammatory proteins, TNF/TNFR superfamily members were dramatically enhanced in HAdV pneumonia patients compared with control subjects. The TNF superfamily consists of 19 structurally related ligands, each binding to one or more of the 29 members of the TNF receptor superfamily (9). It is well known that the receptor-ligand systems of TNFSF/TNFRSF are highly conserved in almost all mammalian cells, and critically involved in various biological events, including host inflammation, programmed cell death, immune cell proliferation and differentiation (26). Our Luminex immunoassay data showed that most TNF/TNFR superfamily members (TNFSF12, TNFSF13/13B, TNFSF14, TNFRSF8, sTNF-R1, sTNF-R2) were enhanced in the BAL, whereas only TNFSF13B, TNFSF14 and sTNF-R1 were dramatically enriched in the plasma of HAdV pneumonia patients. The different TNFSF/TNFRSF expression patterns in BAL and plasma samples indicate that local inflammatory response is more potent than systemic inflammation in children with HAdV pneumonia. However, for pediatric patients, it is more easily to get plasma rather than BAL. Thus, identification of specific systemic inflammatory biomarkers has important value for clinical diagnosis of childhood HAdV pneumonia, especially the severe cases.

Currently, no specific therapy has a demonstrated efficacy in the clinical treatment of pediatric HAdV pneumonia (2), including antiviral drugs. Therefore, all the patients involved in the present study were not treated with antiviral drugs. And all the specimens as well as clinical examination were collected or performed at the acute stage of infection, before corticosteroid treatment. Therefore, in the present study, the use of drugs didn’t affect the dosages. For each patient, we collected the plasma and BAL samples at the same time, to compare the systemic vs local inflammatory response. In the future, we will design a study to collect more longitudinal plasma samples to explore the dynamic change of systemic inflammatory mediators during the course of HAdV pneumonia.

Clinically, about 20% of hospitalized patients with HAdV pneumonia develop to severe cases, of which 25% have severe sequelae. However, it is hard to predict the development or progression of severe HAdV pneumonia at an early period, because sensitive and specific indicators remain unexplored. In the present study, protein levels of TNFSF13B and TNFSF14 were dramatically increased in both plasma and BAL fluid of severe, and featured as key molecules in local and systemic inflammatory network. Moreover, their plasma levels are positively correlated with the CRP levels. These data together indicated that TNFSF13B and TNFSF14 played an important role in host inflammation and disease progression of HAdV pneumonia.

TNFSF13B is mainly produced by adaptive immune cells, in particular B and T lymphocytes, and plays an important role in B cell development (27). TNFSF13B expression is also detected in stromal cells like pulmonary epithelial cells as well as activated neutrophils and macrophages (9, 20). TNFSF13B can bind to three receptors, including the B-cell activation factor receptor (BAFF-R), transmembrane activator and calcium modulator cyclophilin ligand interactor (TACI) and B cell maturation antigen (BCMA) (9). All of these receptors for TNFSF13B are expressed not only on B and T lymphocytes but also on antigen presenting cells, indicating that TNFSF13B’s function extends beyond that of B cell biology. Substantive evidence has demonstrated that TNFSF13B levels are elevated in the BAL of infants with severe RSV infection, and in upper airway secretions from children with human metapneumovirus, influenza virus (H1N1), bocavirus, rhinovirus, RSV, or *Mycoplasma pneumoniae* infections *(* 18). However, the expression and function of TNFSF13B in pneumonia with HAdV infection remains unknown.

TNFSF14 functions as a costimulatory factor to activate lymphocytes and induces pro-inflammatory gene expression *via* activating NF-κB (28). Studies have demonstrated that TNFSF14 plays a broader role in mucosal and systemic immunity, and is linked to dysregulated mucosal function (29). Increased serum TNFSF14 concentrations are detected in patients with pulmonary arterial hypertension. It is well known that TNFSF14 is expressed on various cell types such as T cells, dendritic cells, macrophages, NK cells, and human lung fibroblasts (16). Activation of TNFSF14 signaling leads to NF-κB activation and expression of various downstream proinflammatory mediators (13). In patients with HAdV55 infection, excessive level of TNFSF14 in neutrophils and macrophages is also closely related to lung inflammation and pathological damage (16). Also, in patients with severe asthma, higher sputum TNFSF14 concentrations is correlated with the most impaired lung function (30). Therefore, we believe that the up-regulation of plasma TNFSF13B and TNFSF14 may be a sensitive systemic inflammatory indicator of mucosal and pulmonary infections.

To be noted, most of the severe cases involved in the cohort study were confirmed as HAdV-3 (7.70%) and HAdV-7 (more than 70%) infection. Studies have analyzed the epidemiological and clinical features of pediatric pneumonia in Chongqing of China, and found that among 208 hospitalized children with HAdV pneumonia, HAdV-7 (104, 50.0%) and HAdV-3 (78, 37.5%) were the two major types, followed by HAdV-1, HAdV-55 and HAdV-14, and 80% of patients with single HAdV infections (87, 41.8%) were HAdV-7 infections (31). It has been reported that gene expression of IL-1 family members (IL18, IL36b, IL17rc and IL1a) and TNF superfamily members (TNFSF10, TNFSF11, TNFSF14 and TNFSF15) in peripheral blood mononuclear cells are closely correlated with the severity of HAdV-55 infection (31). Whereas in our study, the plasma levels of most IL1 family members were extremely low or not detected using multiplex assay (data not shown), therefore could not be used as a systemic marker. More importantly, ROC analysis indicated that plasma TNFSF13B and TNFSF14 displayed a great potential to predict severe HAdV pneumonia, no matter which HAdV subtypes are involved.

Overall, our study demonstrated that TNFSF/TNFRSF system played an essential role in both local and systemic inflammatory response in pediatric HAdV pneumonia, and TNFSF13B and TNFSF14 may function as potential systemic inflammatory markers of severe cases of pediatric HAdV pneumonia.

## Data Availability Statement

The original contributions presented in the study are included in the article/supplementary materials; further inquiries can be directed to the corresponding authors.

## Ethics Statement

This study was conducted in accordance with the declaration of Helsinki and approved from the Ethics Committee of Guangzhou Women and Children’s Medical Center, Guangzhou Medical University. The study was conducted in accordance with the Strengthening the Reporting of Observational Studies in Epidemiology (STROBE) guidelines. The patients/participants provided their written informed consent to participate in this study.

## Author Contributions

HF, BL, and CC performed the experiment and wrote the manuscript. DY and LH collected the data and specimens. TD and HL analyzed the data. GL and MW performed the study design and critical revision. All authors contributed to the article and approved the submitted version.

## Funding

This work was funded by Guangzhou Basic Research Projects from Guangzhou Municipal Science and Technology Bureau (No. 202002030061).

## Conflict of Interest

The authors declare that the research was conducted in the absence of any commercial or financial relationships that could be construed as a potential conflict of interest.
